# Analysis of pairwise correlations in multi-parametric PET/MR data for biological tumor characterization and treatment individualization strategies

**DOI:** 10.1007/s00259-016-3307-7

**Published:** 2016-02-13

**Authors:** Sara Leibfarth, Urban Simoncic, David Mönnich, Stefan Welz, Holger Schmidt, Nina Schwenzer, Daniel Zips, Daniela Thorwarth

**Affiliations:** Section for Biomedical Physics, Department of Radiation Oncology, University Hospital Tübingen, Tübingen, Germany; Department of Radiation Oncology, University Hospital Tübingen, Tübingen, Germany; Department of Diagnostic and Interventional Radiology, University Hospital Tübingen, Tübingen, Germany; Faculty of Mathematics and Physics, University of Ljubljana, Ljubljana, Slovenia; Jozef Stefan Institute, Ljubljana, Slovenia

**Keywords:** Integrated PET/MR, Head and neck cancer, Treatment individualization, Multiparametric functional imaging, Correlation analysis, Hypoxia imaging

## Introduction

Biological tumor characterization based on functional and molecular imaging might be highly valuable for radiotherapy (RT). On the one hand, it could allow for an improved target volume definition and an individualized dose prescription within the tumor according to local biological characteristics. Such dose painting strategies can be readily applied with the technical availability of intensity modulated RT (IMRT). Moreover, functional imaging might be of high value for early response assessment and potential treatment adaptation in the course of fractionated RT [1, 2]. Other fields of application are the assessment of chemotherapy and the application of targeted agents, such as hypoxia-sensitizing or antiangiogenic drugs [3, 4].

Both positron emission tomography (PET) and magnetic resonance imaging (MRI) may provide functional information beneficial for personalized treatment strategies. PET imaging using [ ^18^F]-fluorodeoxyglucose (FDG) can be used to monitor glucose metabolism, whereas the hypoxic status of the tumor can be assessed using dedicated tracers such as [ ^18^F]-fluoromisonidazole (FMISO). Diffusion weighted MRI (DW-MRI) provides the possibility to quantify the diffusion of water molecules, which is related to cellular density [5]. Dynamic contrast-enhanced MRI (DCE-MRI) yields a temporally varying signal due to the distribution of contrast agent in blood pool and tissue. By compartmental modeling estimates of quantitative physiological parameters can be derived [6].

With the advent of combined PET/MR imaging [7, 8] the acquisition of simultaneous, intrinsically registered PET and MR data has become possible. This facilitates the comparison and combined analysis of PET- and MR-derived functional imaging data. Simultaneous PET/MR may thus be of high potential for treatment individualization [9, 10].

Recent studies have associated different functional imaging information with RT outcome for head and neck (HN) cancer. This applies to FDG-PET [11, 12], static as well as dynamic FMISO-PET [13–16], apparent diffusion coefficients (ADCs) inferred by DW-MRI [17], as well as DCE-MRI [18, 19]. These studies provide a rationale to adapt RT treatment plans according to functional imaging information.

It is not clear yet if datasets from different functional imaging modalities are completely complementary, or if information is to some extent redundant. Initial analyses of correlations between different functional datasets have already been performed in recent studies. The studies of Rajendran et al. [20] and Thorwarth et al. [21] revealed good voxel-by-voxel correlation of FDG and FMISO in some HN tumors, whereas others showed no clear correlation. The biological basis of the observed correlations may be the hypoxia-inducible factor 1 *α* (HIF 1 *α*) [20]. Similar results were obtained by Zegers et al. [22] comparing uptake of FDG and the hypoxia PET tracer [ ^18^F]-HX4 in patients with non–small cell lung cancer. Houweling et al. [23] quantified correlations between FDG and ADC maps of HN tumors on a voxel level, and found a negative correlation in most patients. Both Newbold et al. [24] and Donaldson et al. [25] found correlations between hypoxia derived from pimonidazole staining and DCE-derived parameter maps on a region-of-interest (ROI) level. A study by Jansen et al. [26] found that neck nodal metastases with positive FMISO uptake differed significantly in median *K*^trans^ values from those with no FMISO uptake.

Earlier studies have shown that a dynamic imaging protocol may be superior compared to a single time frame for hypoxia quantification using FMISO-PET [16]. However, in addition to a late static scan several hours post injection (p.i.), such a dynamic protocol requires a PET acquisition during tracer wash-in in the first minutes p.i. [27], which may hamper its usage in clinical routine. A positive correlation result between early FMISO and DCE information would potentially provide the possibility to infer early FMISO information from DCE, which would facilitate its clinical usage.

To address the question if available functional information of PET/MR is complementary or to some extend redundant, this study extends beyond existing studies by considering a comprehensive set of functional data. Correlations of various functional datasets are quantified on a voxel as well as on a regional level within HN tumors by means of the Spearman correlation coefficient. For the analysis, FDG-PET, FMISO-PET acquired in the wash-in, as well as in the retention phase, ADC maps extracted from DW-MRI, and DCE-MRI derived maps are taken into account. The study is a first explorative, hypothesis generating approach to investigate the utilization of integrated PET/MR for personalized treatment strategies.

## Material and methods

### Patient data

Datasets from 15 HN cancer patients from two different studies were available in total, examined with combined PET/MR (Biograph mMR, Siemens Healthcare, Erlangen, Germany) and PET/CT (Biograph mCT, Siemens Healthcare, Erlangen, Germany) before the start of RT. The studies were approved by the local ethics committee. All patients gave written informed consent for participating in the imaging studies.

For 7 patients (group A) the PET/MR imaging session was performed about 2 h (120–166 min, median: 129 min) after injection of FDG (320–388 MBq, median: 357 MBq). The other 8 patients (group B) were imaged 0–40 min after injection of FMISO (165–377 MBq, median: 339 MBq) in PET/CT using a dynamic acquisition mode, with a subsequent PET/MR imaging session about 3 h p.i. (164–206 min, median: 174 min). For these patients, an additional FDG-PET/CT (307–354 MBq, median: 330 MBq) acquired 1–30 days earlier (median: 8 days) at about 1 h p.i. (55–81 min, median: 71 min) was also available. An overview of the patient cohort including the imaging data available for each patient is shown in Table 1.

**Table 1:** Patient characteristics and acquired datasets

			Tumor	volume of	imaging	DCE-	DW-	# samples	
Patient	Gender	Age	localization	GTV [cm ^3^]	modalities ^*a*^	MRI ^*b*^	MRI ^*c*^	voxel level	regional level
1	m	70	Hypopharynx	35	A	-	x	453	7 ^+^
2	f	57	Oropharynx	41	A	-	x	2519	24
3	m	62	Larynx	16	A	-	x	1050	11
4	m	64	Oropharynx	19	A	-	x	1172	13
5	m	44	Cervical lymph node	19	A	-	x	1176	12
6	f	77	Nasopharynx	16	A	-	x	1005	12
7	m	52	Hypopharynx	16	A	-	x	743	6 ^+^
8	f	62	Base of tongue	16	B	x	(x) ^∗^	1008	13
9	f	57	Oropharynx	23	B	x	x	423	21
10	m	48	Oropharynx	33	B	x	(x) ^∗^	2112	30
11	m	56	Hypopharynx	50	B	x	x	3148	45
12	m	64	Oropharynx	167	B	-	x	10462	166
13	m	57	Oropharynx	10	B	-	x	638	6 ^+^
14	f	55	Oropharynx	56	B	x	x	3556	52
15	f	53	Oropharynx	32	B	-	x	2005	27

^*a*^ A: FDG-PET/MR 2 h p.i., B: FDG-PET/CT 1 h p.i. + dynamic FMISO-PET/CT 0–40 min p.i. + FMISO-PET/MR 3 h p.i., ^*b*^ x: DCE-MRI acquired, -: no DCE-MRI available, ^*c*^ x: ADC-map acquired and evaluated, (x) ^∗^ ADC-map omitted due to spatial distortions (according to visual assessment), ^+^ not used for evaluation due to small sample size

PET images obtained from PET/MR were reconstructed to a voxel size of 2.8×2.8×2.0 mm^3^ using an OSEM 3D algorithm with 2 iterations and 21 subsets (2i21s) and a 3D Gaussian filter of 4 mm. MR-based PET attenuation correction was performed by a vendor-provided segmentation approach based on spoiled gradient-echo sequences with DIXON-based fat-water separation [28]. FMISO-PET images from the PET/CT were reconstructed to a voxel size of 4.1×4.1×5.0 mm^3^ using OSEM 3D with 4i8s and a 3D Gaussian filter of 5 mm. FDG-PET images from the PET/CT were reconstructed to a voxel size of 2.0×2.0×3.0 mm^3^ using OSEM 3D with 3i24s and a 3D Gaussian filter of 3 mm.

MRI acquisitions at the Biograph mMR were performed with the standard 16 channel head neck coil. An anatomical, transversal T2-weighted acquisition using a short time inversion recovery (STIR) sequence was acquired for each patient (repetition time (TR)/echo time (TE)/inversion time (TI) = 4830 ms/37 ms/220 ms, flip angle 160 ^∘^, voxel size 0.7×0.7×4.8 mm^3^, bandwidth 220 Hz/px, 2 averages, acquisition time 3m58s).

In addition, DW-MR images were obtained using a single-shot spin-echo echo-planar imaging (TR/TE = 7400 ms/49 ms, *b*-values 50 s/mm ^2^ and 800 s/mm ^2^, bandwidth 2083 Hz/px, voxel size 2.1×2.1×5.0 mm^3^, 3 averages, spectral attenuated inversion recovery fat suppression, acquisition time 2m26s). ADC maps were obtained from the scanner software (Syngo MR B18P).

For *N*=5 patients also DCE-MR datasets were obtained. An axial T1-weighted fast spoiled gradient echo sequence (TWIST, TR/TE = 2.86 ms/1.01 ms, flip angle 12 ^∘^, voxel size 1.1×1.1×4.0 mm^3^, temporal resolution 2.9 s, bandwidth 530 Hz/px, acquisition time 4m18s) was performed after an automatic fast bolus injection of 0.1 mmol Gd-DTPA per kg patient weight, followed by a saline flush. The field of view included the entire tumor and the common carotid arteries.

For the derivation of the native longitudinal relaxation times needed for DCE-quantification, additional VIBE sequences were acquired with two different flip angles (*α*_1_=2 ^∘^,*α*_2_=12 ^∘^) before contrast agent injection (TR/TE = 4.04 ms/1.52 ms). The image grid was identical to the one of the DCE-MR acquisitions.

### Calculation of parameter maps

The activity of the dynamic FMISO-PET datasets acquired during tracer wash-in was integrated for each voxel between 0 to 4 min p.i. using the rectangle method. By normalizing with respect to the acquisition time range of 4 min, a map of mean activity, $\overline {A}_{\text {FMISO}}$, was obtained. For DCE images, the time-dependent signal enhancement of each voxel was calculated by subtraction of the mean signal before contrast agent injection. Subsequently, maps of mean signal enhancement $\overline {\Delta S}_{\text {DCE}}$ from 0 to 4 min p.i. were calculated analogous to the $\overline {A}_{\text {FMISO}}$ maps.

Before compartmental analysis, DCE images were resampled to the FMISO image grid from PET/MR. Signal-time-curves of DCE were fitted with an in-house implemented software (Matlab R2014b) using the extended Tofts model [29] and the Levenberg-Marquardt least squares algorithm. The arterial input function (AIF) was derived for each patient independently from a fit of the signal-time curve in the common carotid artery. Parameter maps of the volume fraction of the extracellular-extravascular space (EES), *v*_e_, the volume fraction of the blood plasma, *v*_p_, and the volume transfer rate from plasma to the EES, *K*^trans^, were obtained. For regional analysis (see below) compartmental analysis was performed separately on the regional level of 3×3×4 voxels of the resampled DCE images.

### Image registration and tumor volume delineation

For performing the correlation analysis, the GTV of each patient from group A was delineated by an experienced radiation oncologist based on combined information of the FDG-PET and the T2-weighted STIR image [30]. ADC maps were resampled to match the FDG image grid with b-spline interpolation of 3rd order using the Insight Segmentation and Registration Toolkit (ITK version 4.5.2, www.itk.org).

For delineation of the GTVs of group B, manual contours defined by an experienced radiation oncologist on corresponding planning CTs were transferred to the PET/MR datasets by deformable registration of the CT and STIR images. The registrations were performed with elastix [31] using a b-spline parametrized transform and mutual information as similarity measure. Details of the used deformable registration algorithm and the respective parameter set are given in [32]. ADC images from PET/MR were resampled to match the FMISO image grid. Additionally, the FDG image from PET/CT was transformed to the FMISO image grid from PET/MR by deformable registration of the corresponding CT and STIR images with the method described above. The $\overline {A}_{\text {FMISO}}$ map was registered to the PET/MR dataset in the same way.

### Statistical analysis

Correlation analysis was performed for all available pairwise combinations of functional and parametric maps with Python 2.7.6 using the SciPy library (www.scipy.org). Correlations were quantified by evaluating for each patient seperately the Spearman correlation coefficient on a voxel as well as on a regional level within the GTV. For voxel-based analysis, samples were defined by the PET image grid from PET/MR. For regional analysis, samples were defined as averages from non-overlapping sub-regions of the GTV. Each sub-region was defined over 3×3×4 voxels of the PET image grid, corresponding to a size of 8.4×8.4×8 mm^3^. Patients with less than ten subregions were excluded from regional analysis (cf. Table 1).

## Results

**Fig. 1:**
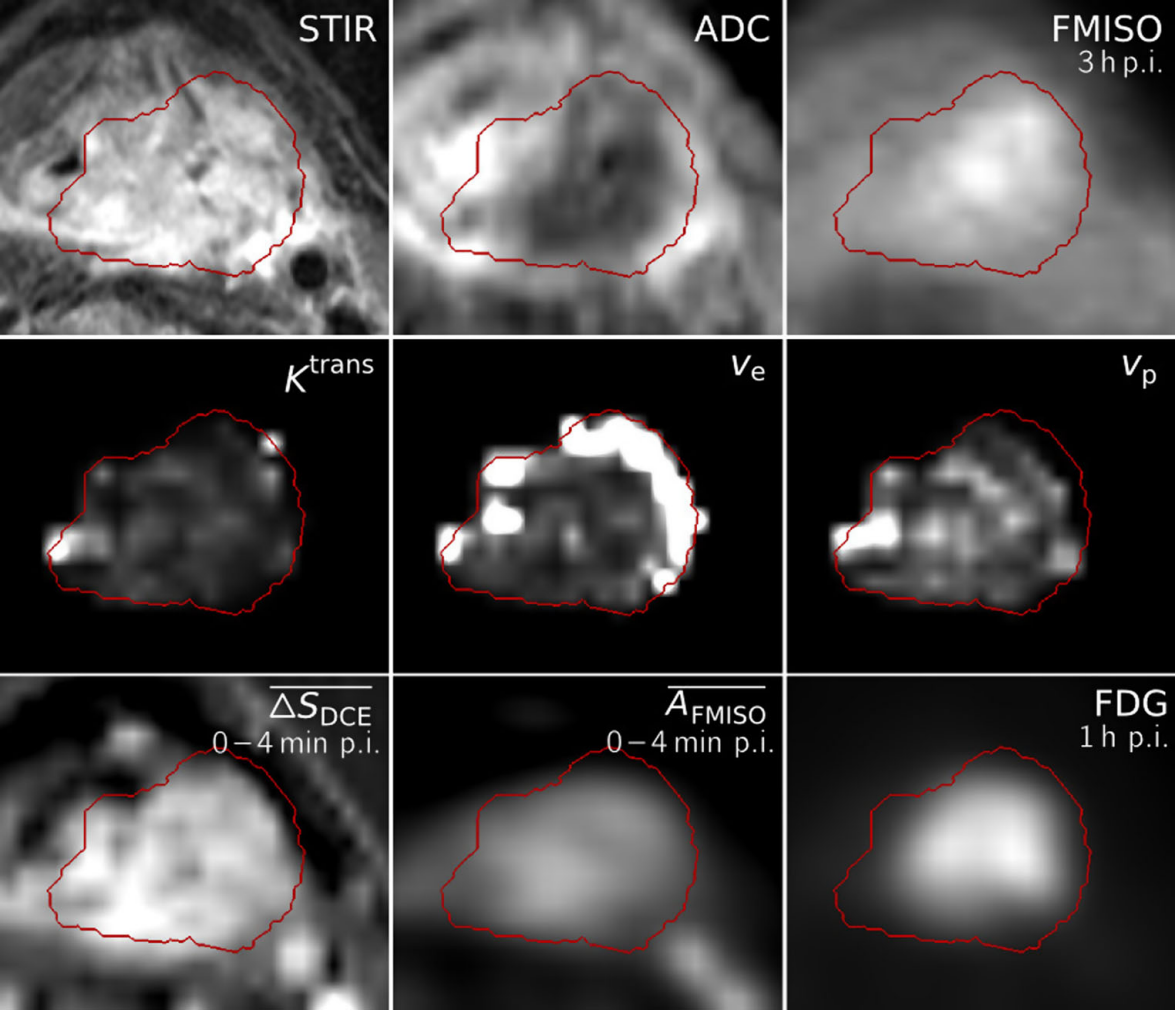
Dataset of Patient 11, showing transversal slices of the anatomical T2-weighted image (STIR), ADC map, FMISO image at 3 h p.i., the DCE-derived maps *K* ^trans^, *v*_e_ and *v*_p_, the maps of mean signal enhancement $\overline {\Delta S}_{\text {DCE}}$ and mean FMISO activity $\overline {A}_{\text {FMISO}}$ in the time range of 0–4 min p.i, and the FDG image at 1 h p.i. All images and parameter maps were acquired in a single PET/MRI session, except for the FDG image and the $\overline {A}_{\text {FMISO}}$ map which were transferred to the PET/MR dataset by deformable registration. The delineation of the GTV is shown in red

All functional images and parameter maps used for pairwise correlation analysis, together with the anatomical STIR acquisition and the delineated GTV, are exemplarily visualized for Patient 11 in Fig. 1.

**Fig. 2:**
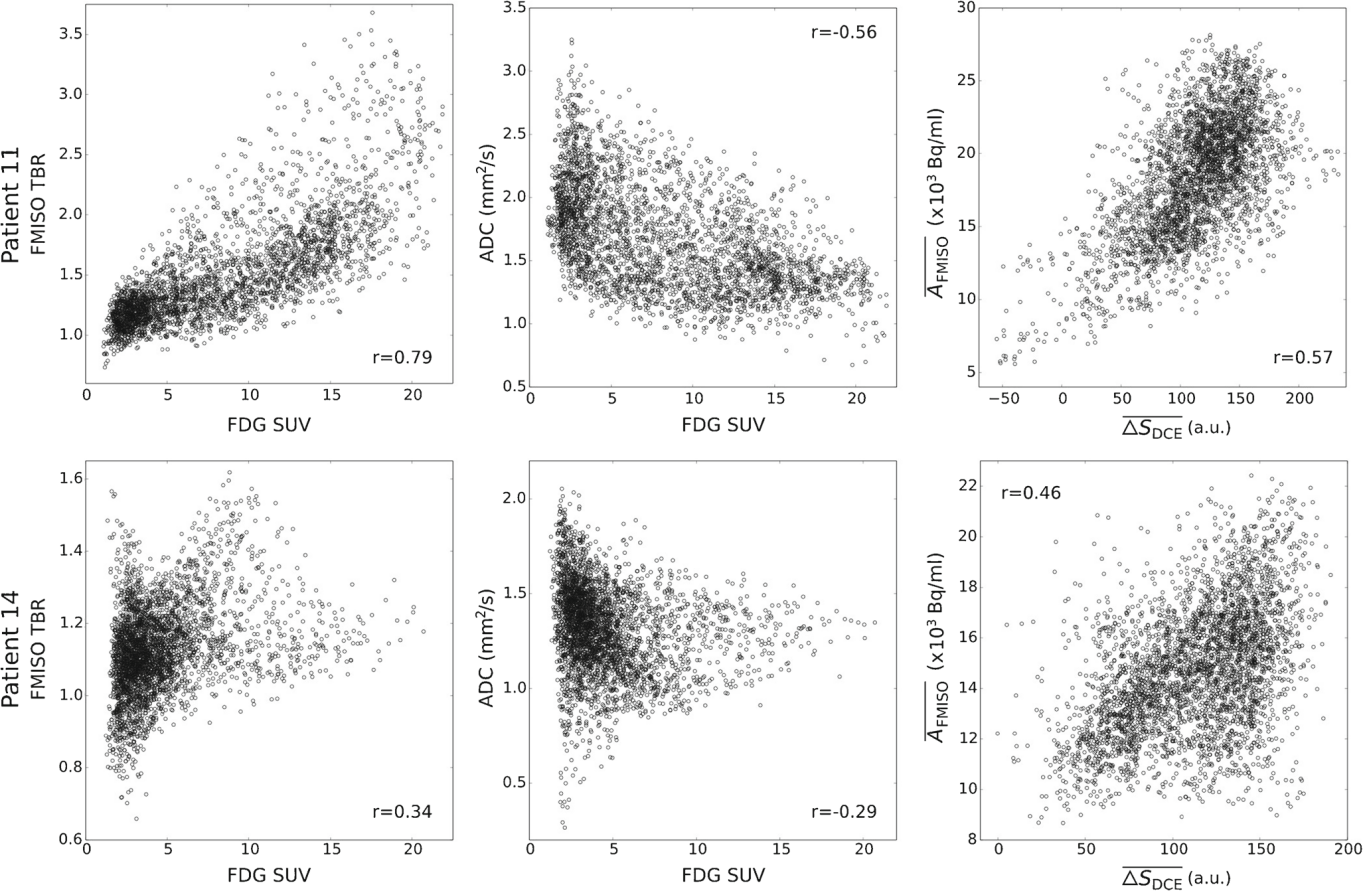
Exemplary scatter plots for Patients 11 (**top**) and 14 (**bottom**), with samples obtained on the voxel level. For increased comparability across patients, FDG activity concentrations were converted to standardized uptake values (SUVs). FMISO data was normalized by devision by the background signal in a deep neck muscle, resulting in the tumor to background ratio (TBR). The Spearman correlation coefficients *r* associated with the scatterplots are shown within each plot

Exemplary scatter plots of the voxel-based pairwise correlation analysis are shown in Fig. 2, visualizing results of two exemplary patients. Scatter plots and corresponding correlation coefficients show that there were patients for which pairs of functional data which showed rather strong correlations (e.g. FDG/ADC, $\overline {\Delta S}_{\text {DCE}}$/$\overline {A}_{\text {FMISO}}$ for Patient 11), while for other patients the correlations of the same pairs were much lower (cf. Patient 14).

**Fig. 3:**
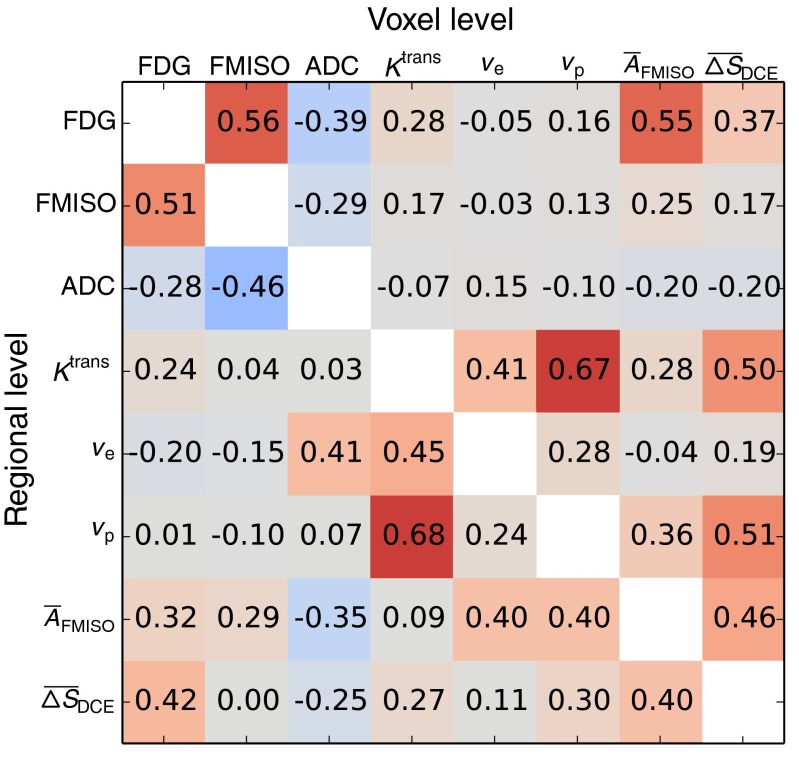
Correlation matrix showing for each pair of functional information the median Spearman correlation coefficients obtained over all respective patient datasets. The upper right triangle shows coefficients derived on the voxel level, whereas the lower left triangle shows the coefficients derived on the regional level

Figure 3 shows a correlation matrix representing the median Spearman correlation coefficients obtained over the available patient datasets for all pairwise combinations of functional data, both for voxel-based and regional analysis. Highest inter-modality median coefficients of the voxel-based analysis were obtained for the combinations FDG/FMISO (*r*=0.56, range: 0.08–0.80, *N*=8), FDG/$\overline {A}_{\text {FMISO}}$ (*r*=0.55, range: 0.19–0.76, *N*=8), $\overline {A}_{\text {FMISO}}$/$\overline {\Delta S}_{\text {DCE}}$ (*r*=0.46, range: 0.30–0.57, *N*=5), and ADC/FDG (*r*=−0.39, range: -0.82–0.30, *N*=13). For regional analysis, values changed to FDG/FMISO (*r*=0.51, range: 0.06–0.86, *N*=7), FDG/$\overline {A}_{\text {FMISO}}$ (*r*=0.32, range: -0.02–0.61, *N*=7), $\overline {A}_{\text {FMISO}}$/$\overline {\Delta S}_{\text {DCE}}$ (*r*=0.40, range: -0.09–0.61, *N*=5), and ADC/FDG (*r*=−0.28, range: -0.98–0.62, *N*=10).

**Fig. 4:**
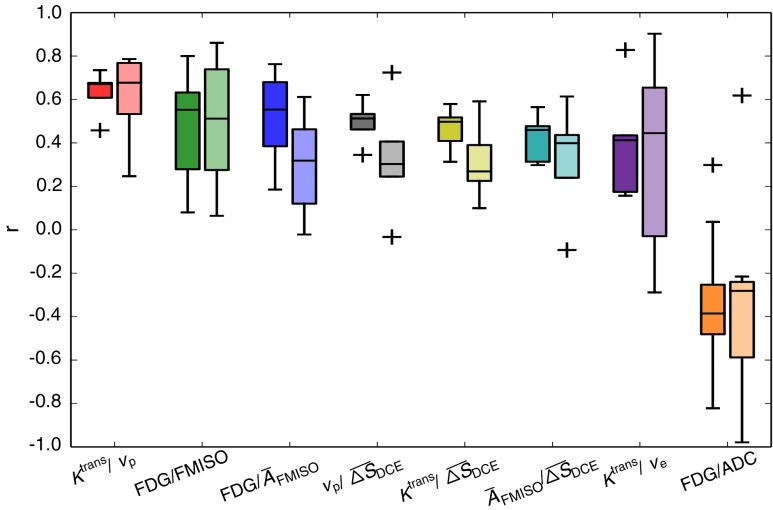
Boxplots showing the inter-patient variation of Spearman correlation coefficients for the pairs with the median voxel correlations according to Fig. 3. For each functional pair, the results from voxel-based analysis (*dark color, left boxes*) and from regional analysis (*light color, right boxes*) are shown

**Fig. 5:**
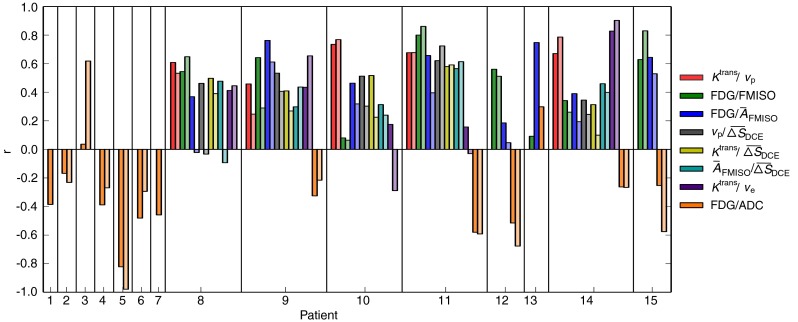
Spearman correlation coefficients obtained for the individual patients. Color encoding is the same as in Fig. 4, with dark and light colors showing the results from voxel-based and regional analysis, respectively

The inter-patient variation of Spearman correlation coefficients for both voxel and regional analysis are shown in Figure 4 for the pairs of highest median voxel correlations. Moreover, correlation coefficients are shown for each patient individually in Fig. 5.

## Discussion

While weak correlations were observed between some functional imaging modalities in the median over all patient datasets, distinct correlations were only present on an individual basis. This applies both to the voxel-based and the regional analysis. FDG and FMISO showed the largest inter-modality median correlations in our study. However, the respective correlation coefficients varied strongly within the patient cohort. This result is in line with the findings of Rajendran et al. [20] and Thorwarth et al. [21]. Similar to Houweling et al. [23], moderate negative correlations were observed between FDG and ADC, with a pronounced variability across patients. No correlations were observed between FMISO and DCE-derived parameters maps. This is different from Newbold et al. [24], Donaldson et al. [25], and Jansen et al. [26]. However, our study is not readily comparable to the results of these authors, since the first authors quantified hypoxia by means of pimonidazole staining after tumor resection and the latter performed the analysis for neck nodal metastases.

We also found a moderate correlation between $\overline {A}_{\text {FMISO}}$ and $\overline {\Delta S}_{\text {DCE}}$. This indicates that they may be measures of similar physiological parameters. However, according to this first analysis the observed correlation does not seem to be sufficient to infer the early FMISO information during wash-in from the DCE data. Instead of using $\overline {\Delta S}_{\text {DCE}}$ maps directly for correlation analysis, they could have also been converted to maps of contrast agent concentration using native T1 maps derived from the VIBE acquisitions. While this might have a slight impact on correlations quantified with the Spearman coefficient due to the dependency of the relation between signal enhancement and concentration on native T1, the conversion to concentration maps would introduce an additional source of error due to uncertainties in native T1 derivation.

For ADC and *v*_e_ maps, correlations may be expected as ADC is commonly related to the fraction of EES, and *v*_e_ is interpreted as the fraction of EES itself. However, in our study weak correlations are only observed on a regional level. One explanation of missing correlations could be that DCE parameter maps in regions with low vascularization are not reliable due to the weak delivery of contrast agent. However, correlation analysis between ADC and *v*_e_ should be performed with further datasets to provide more representative results.

Some of the highest correlations were found between the DCE-based maps *K*^trans^, *v*_e_, *v*_p_, as well as $\overline {\Delta S}_{\text {DCE}}$. This may be either due to inherently correlated parameter estimates in the extended Tofts model used for data analysis, or due to biological relations between the respective parameters.

The determination of multimodal parameter correlations may be substantially compromised by different factors, such as geometrical inaccuracies associated with imaging techniques and image registration, as well as interpolation errors and statistical uncertainties. Geometrical distortions are particularly present in the ADC maps, which were acquired using EPI sequences. For future acquisitions, ideally sequences which are less prone to geometrical distortions should be used in combination with a method for geometrical distortion correction [33, 34]. Also, since no patient positioning system was used during image acquisition in the combined PET/MR examinations, movement of the patients during image acquisition cannot be excluded a priori. Hardware solutions for effective patient immobilization are currently being developed [35]. Finally, geometrical uncertainties are associated with images that were transferred to the PET/MR datasets by deformable registration, which may lead to a reduction of absolute correlation values [36]. An independent analysis of the errors introduced by the different factors is not possible with realistic patient data. In order to account for geometrical uncertainties, a correlation analysis on a regional level was added to the voxel analysis. Such a regional analysis is more robust with respect to geometrical uncertainties, interpolation errors and image noise, whereas averaging may underestimate existing correlations, and additional statistical uncertainties may be introduced. Both increases and decreases in correlation coefficients compared to the voxel-based analysis were observed. However, similar inter-patient distributions were observed (cf. Fig. 4). As a main result of our study we found large variations of correlation coefficients between patients, which most probably can not be explained by the present inaccuracies alone.

DCE parameter maps were derived with the extended Tofts model. However, model parameters may be misinterpreted for some physiological conditions such as highly vascularized tissues with intermediate flow [37]. For other conditions, the model may not fit the data accurately. Other models with fewer assumptions like the four parameter two-compartment exchange model (2CXM) could be used instead if data quality is sufficient in terms of temporal resolution, signal-to-noise ratio and artifacts [6].

Only a limited number of patients was available in this study, especially with respect to DCE-MRI data. Further evaluation should be performed when more patient data is available.

The results in this study extend the correlation analyses performed in previous studies by considering a comprehensive set of functional data. The present results suggest that the different functional datasets derived from DCE-MRI, DW-MRI, FDG-PET and FMISO-PET provide complementary information. Since all these imaging methods were proven to be prognostic for treatment outcome [11–19], this suggests that each method may be of separate value for the adaptation of treatment strategies. However, only pairwise correlations have been analyzed so far. It appears interesting to elaborate if the information from one functional imaging method could be deduced from a combination of several other functional imaging methods. Such an analysis could in the future be performed with machine learning approaches [38] when more patient datasets are available. On the other hand, one may obtain more coherent correlation results if only subgroups of HN tumors are analyzed, for example patients with equal tumor localization, size and staging.

Analyses exploring a potential redundancy between functional PET/MR data may be of value for RT and other treatment modalities due to several reasons. Firstly, using redundant image data and parameter maps for biologically adapted treatments would unnecessarily increase the number of parameters to be adapted with respect to improved outcome. Thus, the correlation analysis performed in the present study constitutes a first step towards the integration of functional imaging into treatment individualization. Before biologically adapted treatments can be used clinically, a number of additional steps are required, such as the correlation of functional parameters to treatment outcome and a thorough regional failure analysis. Further research is needed to clarify which parameter combination provides accurate information about locoregional control probability. Secondly, functional imaging data may concatenate multiple physiological parameters, and interpretation is not always straightforward. A more detailed understanding of functional images and the parameter maps obtained by post-processing models is necessary [37, 39]. Exploring a potential inter-dependency between different datasets may support the interpretation of functional imaging data. Moreover, present or missing correlations between different datasets could potentially also be associated with biological evidence related to treatment response of individual patients. A more comprehensive picture of these issues would allow for a knowledge-driven treatment adaptation, which would then need to be validated in clinical trials.

## Conclusion

Multiparameteric PET/MR provides a substantial amount of different functional imaging data, which may be highly beneficial for cancer treatment adaptation. The results of our study suggest that the associated datasets provide complementary information, and thus could all be of separate value for defining treatment adaption strategies, as well as for treatment response assessment and follow-up. Results of this correlation study might in the future contribute to the design of individually adapted treatment approaches based on multiparametric functional PET/MR imaging.

## Electronic supplementary material

Below is the link to the electronic supplementary material.

(PDF 231 KB)
