# Supplementary figures and images for: Analysis of pairwise correlations in multi-parametric PET/MR data for biological tumor characterization and treatment individualization strategies
Source: Eur J Nucl Med Mol Imaging. 2016 Feb 13;43:1199–208. doi: 10.1007/s00259-016-3307-7 (PMC4869757; doi:10.1007/s00259-016-3307-7)

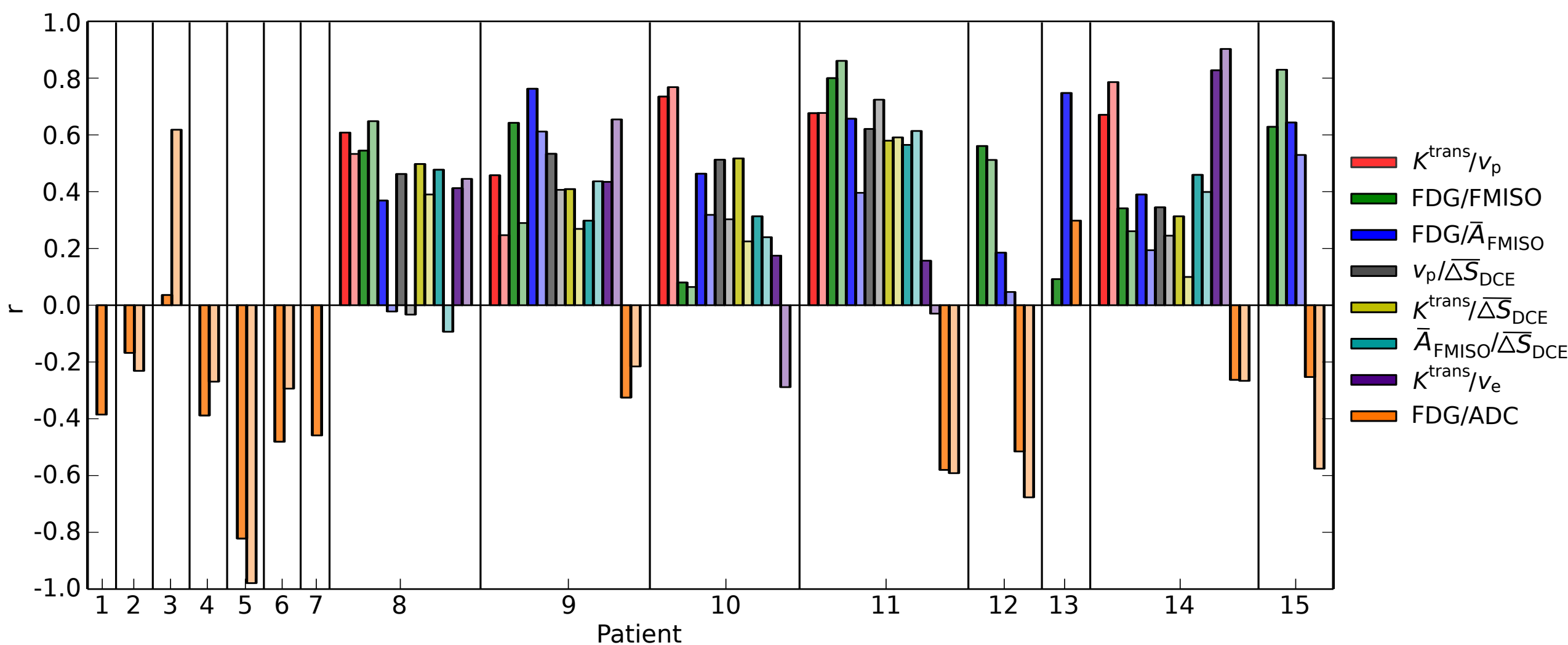

Supplement: Supplementary file 1 — (PDF 231 KB) [file 259_2016_3307_MOESM1_ESM.pdf]
